# Components of *Banisteriopsis caapi*, a Plant Used in the Preparation of the Psychoactive Ayahuasca, Induce Anti-Inflammatory Effects in Microglial Cells

**DOI:** 10.3390/molecules27082500

**Published:** 2022-04-13

**Authors:** Beatriz Werneck Lopes Santos, Daniel Carneiro Moreira, Tatiana Karla dos Santos Borges, Eloisa Dutra Caldas

**Affiliations:** 1 Laboratory of Toxicology, Department of Pharmacy, Faculty of Health Sciences, University of Brasilia, Brasilia 70910-900, Brazil; beatrizwerneck.bia@hotmail.com; 2 Research Center in Morphology and Applied Immunology (NuPMIA), Faculty of Medicine, University of Brasilia, Brasilia 70910-900, Brazil; moreiradc@unb.br (D.C.M.); tatianakarla@unb.br (T.K.d.S.B.)

**Keywords:** ayahuasca, *Banisteriopsis caapi*, ß-carbolines, BV-2 microglial cells, reactive oxygen species, cytokines

## Abstract

*Banisteriopsis caapi* is used to prepare the psychoactive beverage ayahuasca, and both have therapeutic potential for the treatment of many central nervous system (CNS) conditions. This study aimed to isolate new bioactive compounds from *B. caapi* extract and evaluate their biological activity, and that of the known β-carboline components of the plant (harmine, harmaline, and tetrahydroharmine), in BV-2 microglial cells, the in vivo activation of which is implicated in the physiopathology of CNS disorders. *B. caapi* extract was fractionated using semipreparative liquid chromatography (HPLC-DAD) and the exact masses ([M + H]^+^ *m*/*z*) of the compounds in the 5 isolated fractions were determined by high-resolution LC-MS/MS: F1 (174.0918 and 233.1289), F2 (353.1722), F3 (304.3001), F4 (188.1081), and F5 (205.0785). Harmine (75.5–302 µM) significantly decreased cell viability after 2 h of treatment and increased the number of necrotic cells and production of reactive oxygen species at equal or lower concentrations after 24 h. F4 did not impact viability but was also cytotoxic after 24 h. Most treatments reduced proinflammatory cytokine production (IL-2, IL-6, IL-17, and/or TNF), especially harmaline and F5 at 2.5 µM and higher concentrations, tetrahydroharmine (9.3 µM and higher), and F5 (10.7 µM and higher). The results suggest that the compounds found in *B. caapi* extract have anti-inflammatory potential that could be explored for the development of treatments for neurodegenerative diseases.

## 1. Introduction

Ayahuasca is a beverage with psychoactive and medicinal properties prepared mainly by decoction of the plants *Psychotria viridis* and *Banisteriopsis caapi*. Its use by Amazonian natives dates to ancient times and, more recently, it has been used by Christian and shamanic groups [1]. Various studies have shown the potential of ayahuasca and *B. caapi* to treat central nervous system (CNS) disorders, including depression and posttraumatic stress disorder [2,3,4,5,6,7], drug addiction [8,9,10,11,12], Parkinson’s disease [13,14,15], and Alzheimer’s [16]. The β-carboline alkaloids present in *B. caapi* play a major role in the biological activity of the beverage. They inhibit DYRK1A (dual specificity tyrosine-phosphorylationregulated kinase 1A) in cultured neurons, an enzyme that is involved in the pathophysiology of several neurodegenerative diseases [16,17]; increase BDNF levels in the hippocampus of rats [18,19]; and stimulate adult neurogenesis in vitro [20].

**Figure 1:**
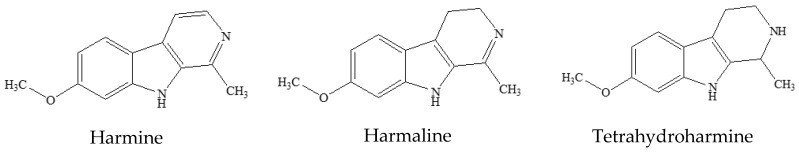
Structures of the main β-carbolines found in *Banisteriopsis caapi*.

The most abundant ß-carbolines in *B. caapi* are harmine, harmaline, and tetrahydroharmine (THH) (Figure 1). Other molecules from this same class have also been detected in the plant, such as harmol, harmalinic acid and other derivatives [21], harmalol [22], and tetrahydronorharmine. Moreover, substances from different groups, such as the proanthocyanidines epicatechin and procyanidin B2, are also present in *B. caapi* [13]. Despite the aforementioned knowledge, very few studies concerning the elucidation of *B. caapi*’s chemical composition have been published, and more research on this plant with tremendous therapeutic potential is still needed.

One of the many cell types in the brain are the nervous system-specific immune cells known as microglia, which work as tissue-resident macrophages [23]. Microglia cells are responsible for several regulatory processes that are crucial for tissue development and homeostasis. These processes include responding to injury and promoting repair, triggering and modulating immune responses to pathogens, and actively performing debris phagocytosis [24,25]. They are present in all regions of the brain and spinal cord, accounting for 12–15% of the total cells found in the CNS [26,27]. Excessive microglial activation and the chronic release of cytokines and other cytotoxic factors occur in many neurodegenerative conditions, including Alzheimer’s and depression, and can be responsible for the neuropathological progression of disease [28,29,30].

Activated microglia are traditionally categorized into two groups based on the displayed phenotype: classically activated M1 and alternatively activated M2. The M1 phenotype is characterized by proinflammatory activity due to the production of mediators, such as interleukin (IL)-6, IL-1β, tumor necrosis factor (TNF), and interferon gamma (IFN-γ). M2 microglia are regarded as anti-inflammatory, based on the secretion of anti-inflammatory factors, including IL-4, IL-13, IL-10, and transforming growth factor (TGF)-β; they contribute to recovery after injury and suppress the proinflammatory response [28,31]. Thus, microglia stand as an attractive therapeutic target. Modulating the pattern of microglial activation might be an effective strategy to prevent or treat neurological diseases [30].

Although some neuromodulator properties of *B. caapi* and its components have been demonstrated, more studies are necessary to elucidate the mechanisms underlying such effects. In this study, the cytotoxicity and anti-inflammatory activity of *B. caapi* extract, selected ß-carbolines (harmine, harmaline, and tetrahydroharmine), and new plant components were investigated using BV-2 microglial cells.

## 2. Materials and Methods

### 2.1. Chemicals and Standards

Analytical standards of harmine (98% purity) and harmaline (95% purity) were obtained from Sigma-Aldrich (St. Louis, MO, USA) and tetrahydroharmine (THH; 95% purity) from LGC Standards (Teddington, UK). Methanol, acetonitrile, and acetic acid, all HPLC grade, were purchased from Merck (Darmstadt, Germany); analytical-grade ammonium acetate was purchased from Neon (São Paulo, Brazil); and formic acid was purchased from Sigma-Aldrich (St. Louis, MO, USA).

### 2.2. Plant Extraction and Spectrometric Analysis

A *Banisteriopsis caapi* sample (type ourinho, containing 6.917 mg/g harmine, 1.583 mg/g harmaline, and 3.084 mg/g THH; [32]) was collected in Goiás, Brazil in 2018. The sample was dried at room temperature for 7 days, powdered by a Wiley mill (Macro Star FT-60, Fortinox, São Paulo, Brazil), sieved through a 500 µm screen, and kept in a plastic bag at room temperature until analysis. Next, 2 g of the sample were sonicated in 40 mL of methanol and macerated for 24 h at room temperature. The extract was filtered through a 0.45 µm syringe filter and diluted 5-fold in purified water prior to the chromatographic separation. Separation was carried out in a semi-preparative 150 × 10 mm Gemini 5u C18 110A column (Phenomenex, Torrance, CA, USA) in an HPLC system coupled with a Shimadzu SPD-M20A Diode Array Detector (DAD). The mobile phase consisted of 50 mM ammonium acetate buffer (pH 4.2) (A) and acetonitrile containing 0.1% (*v*/*v*) acetic acid (B). The gradient was applied as follows: 14 to 22% B 0–15 min; 22 to 25% B 15–20 min; 25 to 100% B 20–21 min; 100 to 14% B 21–26 min; 14% B 26–40 min at a flow rate of 4 mL/min. The injection volume was 1000 µL and detection was set at 230 nm. This wavelength was chosen based on the highest absorption shown by all peaks of interest. Peak fractions were collected in test tubes using a Gilson (FC-203B) fraction collector.

Collected fractions were evaporated in a Centrivap Vacuum Concentrator System (Labconco, Kansas City, MO, USA) and lyophilized (Lyophilizer L101, Liobras, São Paulo, Brazil). The dry fractions were resuspended in methanol with 0.1% formic acid and subsequently analyzed by high-resolution mass spectrometry (UHPLC Ekspert ultra LC 110-XL; Eksigent/Sciex, Washington, DC, USA) coupled with a TripleTOF 5600+ (Sciex, Washington, DC, USA) for exact mass and fragmentation pattern determination.

Stock solutions of isolated fractions and dry *B. caapi* extract at 10.24 mg/mL were prepared by resuspension in ultrapure water. Stock solutions of standards of harmine, harmaline, and THH were prepared in MilliQ^®^ water at 5.12 mg/mL. All stock solutions were then diluted in Dulbecco’s Modified Eagle Medium (DMEM; (Gibco/ThermoFisher, Waltham, MA, USA) in serial dilutions to be used as treatment for cell assays. In all experiments, 4 µL of the treatment solution were used.

### 2.3. Cell Culture

BV-2 microglial cells were obtained from Rio de Janeiro Cell Bank (BCRJ 0356, Rio de Janeiro, Brazil). Cells were cultured in 50 mL cell culture flasks (Kasvi, Brazil) in DMEM supplemented with 10% (*v*/*v*) fetal bovine serum, 1% (*v*/*v*) antibiotics (10,000 units/mL penicillin and 10,000 µg/mL streptomycin) (all from Gibco, Waltham, MA, USA), and 1% (*v*/*v*) non-essential amino acids (M7145, Sigma-Aldrich, St. Louis, MO, USA) at 37 °C and 5% CO_2_ in a humidified atmosphere (incubator MCO-18AC, Sanyo, Japan). Cells were detached with trypsin (Gibco/Thermo-Fisher, Waltham, MA, USA), transferred to a sterile conical tube, centrifuged at 200× *g* for 10 min, and suspended in prepared medium at an appropriate density for each experiment. Cell viability was assessed with trypan blue in PBS (LGC Biotecnologia, Cotia, Brazil).

### 2.4. Viability Assay

The viability of the BV-2 cells after treatment was assayed by CellTiter-Glo^®^ Luminescent Cell Viability Assay (Promega, Madison, WI, USA), which determines the number of viable cells in culture based on quantitation of the ATP. Cells were transferred to 96-well cell culture plates (Greiner bio-one, São Paulo, Brazil) at a density of 50,000 cells/well in 50 µL of the cell suspension and treated with 4 to 512 µg/mL of *B. caapi* extract, fractions F1, F2 (11.4 to 1454 µM), F3 (13.2 to 1690 µM), F4 (21.4 to 2738 µM), and F5 (19.6 to 2510 µM). This concentration range was defined based on a previous test (MTT colorimetric assay, not published) that showed that ayahuasca decreased BV-2 cell viability at concentrations higher than 4 µg/mL. Harmine, harmaline, and THH were tested at concentrations ranging from 9 to 302 µM, which is comparable to the concentration ranges tested using other cell lines [33,34]. Cells with no treatment were diluted in DMEM to serve as control. Plates were incubated for 2 h with 5% CO_2_ at 37 °C and left for 30 min at room temperature prior to the addition of 50 µL of the reagent from the CellTiter-Glo^®^ kit. The plate content was mixed in an orbital shaker for 2 min to induce cellular lysis and then left to stabilize for 10 min at room temperature before luminescence reading in a SpectraMax^®^ Plus 384 luminometer from Molecular Devices (San José, CA, USA).

### 2.5. Apoptosis/Necrosis Assay

Apoptosis/necrosis detection was carried out using an FITC Annexin V/PI kit from BD Biosciences (San Jose, CA, USA). Cells (50 µL) were plated at a density of 100,000 cells/well in DMEM, incubated for 2 h at 37 °C with 5% CO_2_, and the plates washed with phosphate-buffered saline with pH of 7.2 (PBS) at 37 °C. Cells were treated with F2 (5.7 to 727 µM), F4 (10.7 to 1369 µM), and harmine (2.4 to 75.5 µM). DMEM served as a negative control and dimethyl sulfoxide (DMSO) 10% (*v*/*v*) was used as a positive control. Plates were incubated for 24 h at 37 °C with 5% CO_2_ in a humidified atmosphere and then washed with PBS prior to the addition of 50 µL of FITC anexin V/PI. Cell suspension was analyzed by flow cytometry (LSR II Fortessa™, BD Biosciences, Franklin Lakes, NJ, USA), with a total of 10,000 events per sample, using FACSDiva software v 7.0 (BD Biosciences, Franklin Lakes, NJ, USA). Mean fluorescence intensity (MFI) was obtained by processing data in FlowJo^TM^ software v 10.6.1. The concentrations tested in this assay were defined based on the results of the viability test (2 h incubation) and longer incubation time (24 h).

### 2.6. ROS Production

Reactive oxygen species (ROS) were assessed using 6-carboxy-2′,7′-dichlorodihydrofluorescein diacetate (carboxy-H_2_DCFDA) from Invitrogen (Waltham, MA, USA). Cells were plated, treated (1 to 32 µM) as described for the apoptosis/necrosis assay, washed twice with PBS, and incubated with 200 µL of 10 µM carboxy-H_2_DCFDA diluted in DMEM. Treatment concentrations ranged from 2.8 to 90.9 µM for F2, from 5.3 to 171 µM for F4, and from 4.7 to 151 µM for harmine. The fluorescence intensity of carboxy-H_2_DFCDA was assessed by flow cytometry as described in Section 2.5. The concentrations tested in this assay were defined based on the results of the cell viability assays, and on previous studies on the modulatory effects of β-carbolines on ROS production using cell and animal models [35,36].

### 2.7. Cytokine Production

Cytokine production was assessed using a Cytometric Bead Array Mouse Th1/Th2/Th17 Cytokine Kit from BD Biosciences (San Jose, CA, USA). Cells were plated as described for viability and incubated for 2 h at 37 °C and 5% CO_2_ with *B. caapi* extract and fractions F1 to F5 at 0.5–64 µg/mL; and harmine, harmaline, and THH at 0.5–16 µg/mL (2.4 to 75.5 µM). DMEM served as a control. After incubation, the cell culture supernatant was collected and stored at −80 °C until analyses. The test assay was performed according to the manufacturer’s specifications. Briefly, samples were incubated for 2 h with antibody-coated beads (capture beads) and detection antibody (detection reagent). After incubation, the plate was washed once and the complex (capture beads-cytokine-detection antibody) was suspended with kit buffer assay. Flow cytometry was used to evaluate 2500 events/samples using FACSDiva software v7.0 (BD Biosciences, Franklin Lakes, NJ, USA) and data were processed by FCAP Array software V. 3.0 (BD Biosciences, Franklin Lakes, NJ, USA). A standard curve for each cytokine (20 to 5000 pg/mL) was prepared and used for quantitative analysis.

### 2.8. Statistical Analyses

The data are reported as the mean and standard error of the mean (SEM) of at least 3 and a maximum of 6 independent experiments. Experimental differences were tested for statistical significance using one-way analysis of variance (ANOVA) using GraphPad Prism 5 software (San Diego, CA, USA). A *p*-value < 0.05 was deemed as statistically significant and is indicated in the figures and tables by an asterisk. *p*-values < 0.01 and <0.001 are indicated by 2 and 3 asterisks, respectively.

## 3. Results and Discussion

### 3.1. Preliminary Phytochemical Analyses

**Figure 2:**
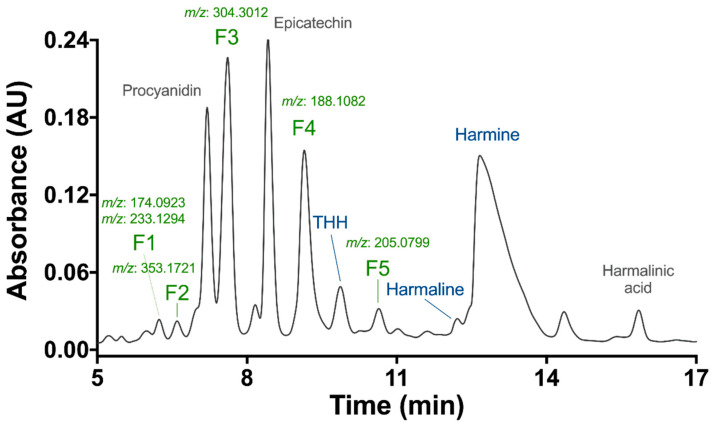
HPLC-DAD chromatogram of a *Banisteriopsis caapi* methanolic extract, at 230 nm. Putative identification of procyanidin and epicatechin (Samoylenko et al., 2010) and harmalinic acid (Hashimoto, 1975). *m*/*z* refers to the protonated ion ([M + H]^+^) in the UHPLC-TripleTOF 5600+. THH: tetrahydroharmine.

HPLC-DAD analysis of the *B. caapi* methanolic extract using a semi-preparative column yielded several peaks, with high absorbance observed at 230 nm (Figure 2). Fractions were collected and analyzed for exact mass determination in the TripleTOF 5600+ instrument. All fractions demonstrated chromatographic pureness with the presence of one peak of high intensity, except for fraction F1, which seemed to contain two compounds with incomplete chromatographic separation. Indeed, F1 showed the presence of two intense masses ([M + H]^+^) of *m*/*z* 174.0923 and 233.1294. The other fractions were F2 (*m*/*z* 353.1721), F3 (*m*/*z* 304.3012), F4 (*m*/*z* 188.1082), and F5 (*m*/*z* 205.0799). To the best of our knowledge, the compounds found in these fractions have not previously been described in *B. caapi* samples. A tentative elucidation of the compound structures was performed by nuclear magnetic resonance (NMR), but the spectra were not conclusive due to the low amount obtained during the purification process.

The exact masses and fragmentation patterns of three collected fractions already described in the literature were putatively identified as procyanidin (*m*/*z* 579.2425), epicatechin (*m*/*z* 291.0868) [13] and harmalinic acid (*m*/*z* 245.0923), first reported by Hashimoto and Kawanishi [21]. The identity of harmine, harmaline, and THH fractions was confirmed by comparing their exact mass and fragmentation information with that of analytical standards (Figure 2). The spectrometric data for all fractions are shown in Supplementary Materials (Figure S1). The concentrations of the fractions used in the treatments (µg/mL) were converted to µM using their respective molecular masses estimated experimentally in this study, except for *B. caapi* (BC) extract (mixture of many compounds) and F1 (mixture of two compounds).

### 3.2. Cytotoxicity Assays—Viability, Apoptosis/Necrosis, and ROS Production

**Figure 3:**
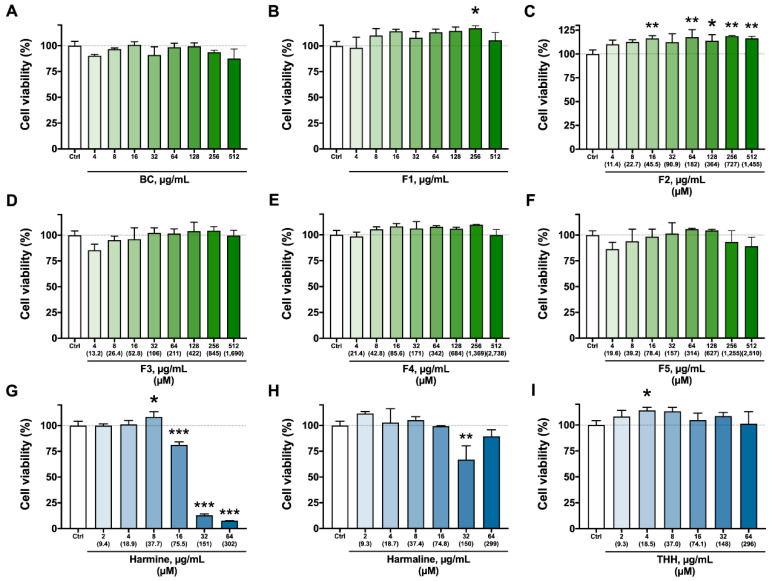
Viability of BV-2 cells treated at different concentrations for 2 h with *Banisteriopsis caapi* extract (BC, (**A**)), the isolated fractions F1 to F5 (**B**–**F**), and β-carbolines (**G**–**I**). Values are presented as mean ± SEM (*n* = 3, except for control, *n* = 6). * *p* < 0.05, ** *p* < 0.01, *** *p* < 0.001 compared to cells with no treatment (control).

**Figure 4:**
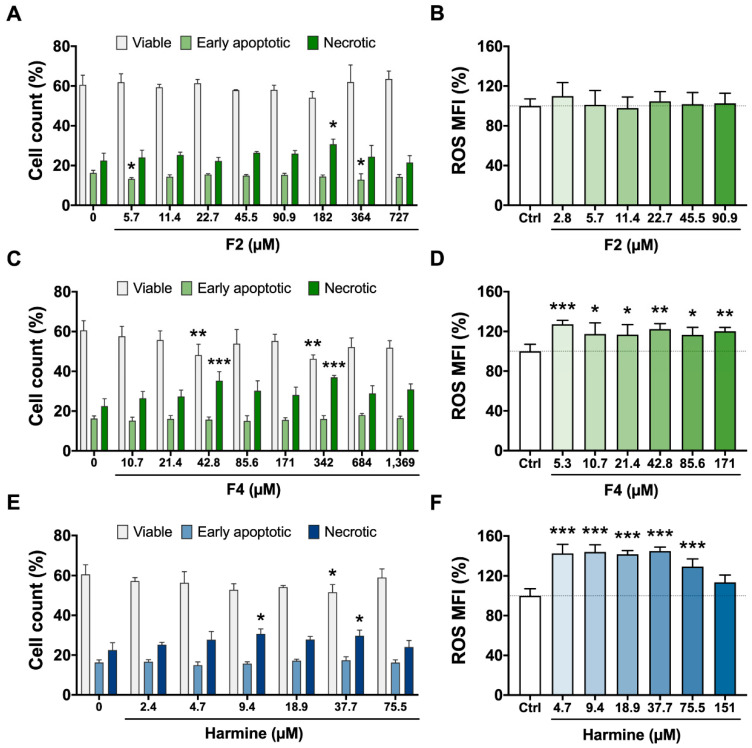
Cell count (left) and ROS production (right) after 24 h in BV-2 cells treated with *Banisteriopsis caapi* extract fractions F2 (**A**,**B**) and F4 (**C**,**D**) and harmine (**E**,**F**). Values are presented as mean ± SEM (*n* = 4–5); * *p* < 0.05, ** *p* < 0.01, *** *p* < 0.001 compared to cells with no treatment (control). Viable + early apoptotic and necrotic cells in all treatments is equal to 100%.

Figure 3 shows the results of the viability assay (ATP quantitation) of BV-2 cells treated for 2 h with *B. caapi* extract, isolated fractions (F1 to F5), and the β-carbolines (harmine, harmaline, and THH). Treatment with *B. caapi,* F3, F4, and F5 did not have any significant impact on cell viability (Figure 3). However, F4 decreased the proportion of viable cells and increased that of necrotic cells at 42.8 and 342 µM after 24 h (Figure 4). The results indicate a cytotoxic effect of F4 on BV-2 cells after 24 h of exposure, although a dose–response relationship was not shown but a plateau pattern. Fractions F1 and F2 appeared to have a stimulative effect on BV-2 cells after 2 h of incubation, as cell viability was significantly enhanced after treatment at 256 µg/mL for F1 and 16 µg/mL (45.5 µM) or higher, except at 32 µg/mL (91 µM) (Figure 3). After 24 h of incubation, F2′s effect seemed to continue, as the proportion of early apoptotic cells decreased at 5.7 and 364 µM, although necrotic cells increased at 182 µM (Figure 4).

Among the β-carbolines, harmine had the highest impact on cell viability after 2 h of treatment, increasing it transiently at 8 µg/mL (37.7 µM), then decreasing it at 16 µg/mL (75.5 µM) and further decreasing it at the 2 highest doses (151 and 302 µM) (Figure 3). Harmaline and THH had opposite results, with a decrease at 32 µg/mL (149 µM) and enhanced activity at 4 µg/mL (18.5 µM), respectively (Figure 3). After 24 h (Figure 4), harmine was cytotoxic at 9.4 and 37.7 µM, as evidenced by the increase in the number of necrotic cells and the decrease in the number of viable cells (only at 37.7 µM). Nakagawa et al. [33] observed that harmine at 250 and 500 µM induced cell death and decreased ATP production in rat hepatocytes 1 to 3 h after the treatment, a result that was also found in the present study at much lower concentrations (down to 75.5 µM). Harmaline also has a negative effect on viability, but this is less pronounced when compared to harmine [33], which was also observed in the present study.

The viability of immortalized rat mesencephalic dopaminergic neurons was shown to decrease significantly after treatment with harmine and THH at 10 µM, and with harmaline at 80 µM [34]. These concentrations are lower than those that impacted cell viability in the present study. This is expected since the neurons were incubated for a much longer period (24 h) than that for microglia cells in the present study (2 h). Furthermore, different cell types might respond differently to the same stimulus. Indeed, harmine reduces the viability of human gastric carcinoma cells treated at concentrations of up to 10 µM for 48 h, whereas treating mouse fibroblasts at the same concentrations does not impact viability [37]. Harmaline (0.5–10 µM) inhibits viability and induces the apoptosis of human liver carcinoma HepG2 cells through upregulation of the p53/p21 and Fas/FasL signaling pathways, an effect that is time and concentration dependent [38]. One mechanism by which *B. caapi* extract, its fractions, and β-carbolines therein might exert their effects is through the modulation of reactive species’ steady state levels.

A significant increase in ROS production was found 24 h after treatment with F4 at all concentrations tested (2.7 to 342 µM) and for harmine from 4.7 to 75.5 µM, reaching control levels at 151 µM (Figure 4). This is probably because at this concentration, most of the cells were unable to function correctly. In both cases, high ROS production followed a plateau pattern, although a slight decrease in the mean level was observed at 75.5 µM after harmine treatment. Reactive oxygen species (ROS) are released by phagocytic cells as a host defense strategy. Excess ROS induce programmed cell death or necrosis, induce or suppress the expression of many genes, and activate cell signaling cascades [39]. Intracellular ROS can influence the cell cycle, and whether they promote or inhibit the process depends on their concentration, type of target cell, extracellular stimuli, and the duration of exposure [40]. Increased ROS production due to harmine has already been described [33]. This effect was attributed to two factors: (i) the depletion of glutathione, an antioxidant tripeptide that plays a pivotal role in redox homeostasis, and (ii) loss of mitochondrial membrane potential. Thus, harmine cytotoxicity has been explained to occur due to mitochondrial damage and oxidative stress [33]. The link between loss of cell viability and oxidative stress after 24 h was also found in the present study for harmine and F4, although the effects were not clearly dose dependent.

### 3.3. Cytokine Production

Most treatments displayed an anti-inflammatory pattern on BV-2 cells, with a reduction in the release of proinflammatory cytokines (IL-6, IL-17, TNF, and/or IFN-γ) and an increase in the release of IL-4 or IL-10, which are anti-inflammatory, probably due to a change in the cell phenotype to an M2 pattern [28,31]. IL-2, which is mainly produced by CD4^+^ T lymphocytes, regulates T cell development and homeostasis, with immunostimulatory or immunoinhibitory activity depending on the target cell [41,42]. Several studies have reported the involvement of IL-2 in the proinflammatory responses of BV-2 cells [43,44]. For example, dexmedetomidine, known for its anti-inflammatory activity in vivo, reverses the inflammatory effects of lipopolysaccharide (LPS) on BV-2 cells via inhibition of the NF-κB signaling pathway and proinflammatory cytokines, including IL-2 [44].

The results of the cytokine release in BV-2 cells treated with *B. caapi* crude extract (BC), and with fractions F1 and F2 are shown in Figure S2 (Supplementary Materials). BC and F1 significantly increased IL-4 production relative to the control at 0.5 and 4 µg/mL, respectively, and F2 significantly increased IL-10 production at the highest concentration of 64 µg/mL (182 µM), indicating an anti-inflammatory effect of the treatments, although the effects were highly variable and did not follow a dose–response pattern.

The results for the treatments with F3, F4, F5, and β-carbolines are shown in Table 1. F3 treatment significantly decreased the production of IL-2 and IL-6 by BV-2 cells at the highest concentration (211 µM), showing an opposite modulation. Treatment with F4 significant decreased the levels of proinflammatory cytokines IFN-γ and TNF only at intermediate doses (10.7 to 85.6 µM for IFN-γ), showing a U-shaped dose response only seen in this treatment. This pattern might be explained by the hormesis paradigm, a dose–response phenomenon characterized by a low-dose stimulation and high-dose inhibition, representing an adaptive response of a biological system, and providing central support for the neuroprotective responses [45]. IL-2 production was down to non-detected levels after F4 treatment (10.7–171 µM), but this decrease was not statistically significant.

IL-6 production was impacted by F4 at concentrations equal or higher than 10.7 µM (except at 171 µM), and at the intermediate doses, IL-6 levels were below the detection limit, also indicating a U-shaped dose response pattern. Treatment with F5 significantly decreased the production of all proinflammatory cytokines by BV-2 cells at low concentrations, starting at 2.5 µM for IL-17A. As the F5 concentration increased, the concentration of these cytokines decreased to levels that could not be detected (Table 1).

Harmine did not exert much of an effect on most cytokines at the tested concentrations, except for a gradual significant decrease in TNF release at the highest doses (18.9 to 75.5 µM) and IL-2 at 18.9 and 75.5 µM (Table 1). In RAW 264.7 macrophages, much lower concentrations of harmine are enough to suppress TNF (5 µM) IL-1β (1 µM) and IL-6 (1 µM) [46]. Moreover, harmine was shown to decrease NO production in LPS-induced macrophages and suppresses inflammation in vivo [46]. The anti-inflammatory property of harmine was attributed to its inhibitory effect on the NF-κB signaling pathway [46], a conclusion that may be extrapolated to the results found in the present study considering the well-known involvement of this pathway in regulating immune and inflammatory responses [47]. Another in vivo study supports the anti-inflammatory activity of harmine, which suppresses inflammatory responses in the serum and kidney of mice [48]. The anti-inflammatory effect was associated with the inhibition of TNF-α, IL-6 and IL-1β secretion, probably through inhibition of the TLR4-NF-κB pathway, and a reduction in the enzymatic production of ROS by myeloperoxidase [48].

Harmaline significantly reduced the release of all proinflammatory cytokines, except IL-6. The reduction in IL-17A, IFN-γ, and TNF levels occurred at the three highest doses while IL-2 production was not detected at all tested concentrations. Harmaline was the only treatment that decreased the release of the anti-inflammatory cytokine IL-10 but only at 18.9 µM. Finally, THH treatment significantly decreased IL-6, TNF, and IFN-γ production at 18.5 µM µg/mL or higher while it increased that of IL-4 at 2.3 µM, although this anti-inflammatory effect was not maintained at higher doses (Table 1).

Noteworthy, isolated fractions F4 and F5 exerted a more prominent anti-inflammatory effect on BV-2 cells than harmine or the *B. caapi* extract. These components might help explain the anti-inflammatory effects of ayahuasca or the plant observed in vivo and in vitro [49,50,51]. In a study with depressive patients, treatment with ayahuasca (1 mL/kg) for 2 days decreased both circulating C-reactive protein levels, which is secreted by the liver during an inflammatory response, and depression symptoms compared with the levels of these parameters prior to intervention, although no impact on circulating IL-6 and BDNF levels was found [49].

The role of excessive microglial activation accompanied by the chronic release of cytokines and other cytotoxic factors in neurodegenerative diseases has been widely documented [28,29,30]. Particularly, inflammation can trigger depressive symptoms and is associated with suicidal behavior in patients treated with IFN-based or IL-2 immuno-therapy [52]. Depression is indeed one of the main neurological conditions for which the therapeutical use of ayahuasca has been investigated, including studies with humans [4,5,7]. However, the mechanisms involved in this therapeutical potential have not been fully elucidated so far [2,7]. If the involvement of cytokine regulation by ayahuasca and its components is confirmed (e.g., blocking the pathways involved in cytokine production and release), the beverage may be an important adjuvant therapy to treat depression and other neurodegenerative conditions.

## 4. Conclusions

This study showed that selected β-carbolines and some of the new components, which had not been previously described, found in *B. caapi* extract have a cytotoxic effect at high concentrations but also exert an important anti-inflammatory effect at low levels in microglial BV-2 cells by decreasing proinflammatory cytokine release. This effect was mainly found for harmaline and THH and the fractions F4 and F5, indicating the importance of the new components in the overall therapeutical potential of ayahuasca for the treatment of many central nervous system conditions described in various studies. Future work should focus on isolating a larger amount of the compounds in the identified fractions to completely elucidate their chemical structures.

To the best of our knowledge, this is the first study to report the effects of substances present in *B. caapi* in modulating the release of pro- and anti-inflammatory cytokines by microglia cells. Such activity warrants further investigation using other cell lines and the measurement of other cytokines involved in inflammatory events.

## Figures and Tables

**Table 1:** Cytokine production by BV-2 cells after treatment for 2 h with *Banisteriopsis caapi* extract fractions F3, F4, and F5 and the β-carbolines harmine, harmaline, and tetrahydroharmine (THH). Values significantly different from controls are shown in bold.

Fraction µM	IL-2 (pg/mL)	IL-4 (pg/mL)	IL-6 (pg/mL)	IL-10 (pg/mL)	IL-17A (pg/mL)	IFN-γ (pg/mL)	TNF (pg/mL)
**Control** (*n* = 6)	3.84 ± 0.96	1.49 ± 0.51	1.43 ± 0.22	1.77 ± 0.44	0.54 ± 0.1	0.88 ± 0.08	7.59 ± 0.81
**F3** (*n* = 3, except for 1.7 and 211 µM, *n* = 6)							
1.7	1.16 ± 0.65	2.57 ± 1.19	0.86 ± 0.86	2.08 ± 1.08	0.19 ± 0.19	0.71 ± 0.11	5.57 ± 0.82
6.6	0.00 ± 0.00	3.86 ± 1.45	0.93 ± 0.43	1.24 ± 0.71	0.42 ± 0.42	0.75 ± 0.15	5.43 ± 1.4
13.2	1.46 ± 1.46	4.61 ± 0.39	1.72 ± 0.23	2.09 ± 1.53	0.49 ± 0.49	0.58 ± 0.15	5.22 ± 0.21
26.4	5.01 ± 1.67	3.81 ± 0.53	0.81 ± 0.17	3.06 ± 1.06	0.00 ± 0.00	0.27 ± 0.14	6.56 ± 0.82
52.8	1.37 ± 1.37	2.93 ± 1.54	1.06 ± 0.15	1.70 ± 1.27	0.29 ± 0.29	0.54 ± 0.13	6.85 ± 0.44
106	0.00 ± 0.00	1.95 ± 1.26	0.58 ± 0.29	0.39 ± 0.39	0.18 ± 0.18	0.41 ± 0.17	5.53 ± 2.16
211	0.00 ± 0.00 *	1.32 ± 0.87	0.39 ± 0.12 *	0.19 ± 0.19	0.01 ± 0.01	0.43 ± 0.13	5.34 ± 0.84
**F4** (*n* = 3, except for 1.7 µM, *n* = 6)							
2.7	2.93 ± 1.11	2.15 ± 1.1	0.88 ± 0.08	1.61 ± 0.57	0.54 ± 0.16	0.55 ± 0.09	6.83 ± 0.73
10.7	0.00 ± 0.00	2.66 ± 1.5	0.60 ± 0.3 *	1.44 ± 0.81	0.00 ± 0.00	0.28 ± 0.25 *	4.24 ±
21.4	0.00 ± 0.00	0.60 ± 0.60	0.00 ± 0.00 ***	0.43 ± 0.43	0.00 ± 0.00	0.08 ± 0.08 **	4.42 ± 0.84
42.8	0.00 ± 0.00	1.04 ± 1.04	0.00 ± 0.00 ***	0.00 ± 0.00	0.16 ± 0.16	0.03 ± 0.03 ***	2.01 ± 1.08 **
85.6	0.00 ± 0.00	2.70 ± 1.64	0.25 ± 0.25 **	0.00 ± 0.00	0.32 ± 0.32	0.15 ± 0.15 **	2.68 ± 1.36 *
171	0.00 ± 0.00	3.96 ± 0.91	0.89 ± 0.11	1.16 ± 0.35	0.12 ± 0.12	0.65 ± 0.07	6.80 ± 1.01
342	0.30 ± 0.30	3.03 ± 0.11	0.23 ± 0.11 **	0.14 ± 0.14 *	0.00 ± 0.00 ±	0.61 ± 0.12	4.04 ± 0.22
**F5** (*n* = 3, except for 2.5 µM, *n* = 5)							
2.5	2.43 ± 1.02	1.93 ± 0.97	0.54 ± 0.15	1.27 ± 1.21	0.09 ± 0.09 **	0.57 ± 0.09	5.67 ± 0.48
9.8	0.95 ± 0.95	3.44 ± 1.08	0.88 ± 0.61	1.32 ± 0.42	0.00 ± 0.00 **	0.67 ± 0.14	8.47 ± 2.51
19.6	0.00 ± 0.00 *	2.28 ± 1.32	0.49 ± 0.12	0.46 ± 0.46	0.17 ± 0.09	0.52 ± 0.19	3.99 ± 0.21
39.2	0.00 ± 0.00 *	1.95 ± 0.2	0.09 ± 0.09 **	0.00 ± 0.00	0.00 ± 0.00**	0.16 ± 0.10 **	1.81 ± 0.14 **
78.4	0.00 ± 0.00 *	0.68 ± 0.68	0.00 ± 0.00 **	0.00 ± 0.00	0.00 ± 0.00 **	0.16 ± 0.16 **	1.80 ± 1.13 **
157	0.00 ± 0.00 *	0.00 ± 0.00	0.00 ± 0.00 **	0.00 ± 0.00	0.00 ± 0.00 **	0.00 ± 0.00 ***	0.35 ± 0.35 ***
314	0.00 ± 0.00 *	0.00 ± 0.00	0.00 ± 0.00 **	0.00 ± 0.00	0.00 ± 0.00 **	0.00 ± 0.00 ***	0.01 ± 0.01 ***
**Harmine** (*n* = 6, except for 18.9 µM, *n* = 5)							
2.4	1.23 ± 0.85	2.69 ± 0.64	0.81 ± 0.16	0.84 ± 0.34	0.33 ± 0.13	0.56 ± 0.04	5.77 ± 0.61
9.4	1.84 ± 0.87	1.91 ± 0.90	0.86 ± 0.31	1.18 ± 0.62	0.17 ± 0.09	0.70 ± 0.17	5.15 ± 0.95
18.9	0.29 ± 0.29 *	2.35 ± 0.97	0.72 ± 0.21	0.44 ± 0.23	0.11 ± 0.11	0.55 ± 0.10	4.25 ± 0.51 *
37.7	2.33 ± 0.76	2.51 ± 1.35	1.39 ± 0.27	1.66 ± 0.51	0.51 ± 0.30	0.87 ± 0.14	4.04 ± 0.61 *
75.5	0.20 ± 0.2 *	1.38 ± 0.76	0.63 ± 0.17	1.25 ± 0.75	0.30 ± 0.13	0.78 ± 0.09	3.77 ± 0.61 **
**Harmaline** (*n* = 3, except for 18.7 µM, *n* = 6)							
2.4	0.00 ± 0.00 **	1.32 ± 1.32	0.18 ± 0.18	0.59 ± 0.59	0.26 ± 0.26	0.31 ± 0.31	3.12 ± 3.12
9.3	0.00 ± 0.00 **	1.57 ± 1.23	0.51 ± 0.43	0.73 ± 0.73	0.14 ± 0.14	0.33 ± 0.33	2.91 ± 1.89
18.7	0.00 ± 0.00 **	0.00 ± 0.00	0.48 ± 0.31	0.12 ± 0.12 *	0.02 ± 0.02 *	0.20 ± 0.12 *	1.29 ± 0.74 **
37.4	0.00 ± 0.00**	0.00 ± 0.00	0.00 ± 0.00	0.00 ± 0.00	0.00 ± 0.00 *	0.01 ± 0.01 *	0.15 ± 0.15 **
74.8	0.00 ± 0.00**	0.00± 0.00	0.00 ± 0.00	0.00 ± 0.00	0.00 ± 0.00 *	0.00 ± 0.00 **	0.00 ± 0.00 **
**THH** (*n* = 3)							
2.3	4.25 ± 2.25	6.14 ± 0.47 **	1.52 ± 0.37	3.16 ± 1.10	0.80 ± 0.41	0.77 ± 0.06	6.68 ± 0.38
9.3	0.00 ± 0.00	4.32 ± 1.43	0.00 ± 0.00 **	1.01 ± 1.01	0.39 ± 0.39	0.31 ± 0.17 **	2.58 ± 1.12 **
18.5	0.00 ± 0.00	0.00 ± 0.00	0.00 ± 0.00 **	0.17 ± 0.17	0.00 ± 0.00	0.08 ± 0.08 ***	0.85 ± 0.85 ***
37.0	0.00 ± 0.00	1.45 ± 1.45	0.00 ± 0.00 **	0.00 ± 0.00	0.00 ± 0.00	0.01 ± 0.01 ***	0.72 ± 0.68 ***
74.1	0.00 ± 0.00	0.00 ± 0.00	0.00 ± 0.00 **	0.00± 0.00	0.00 ± 0.00	0.00 ± 0.00 ***	0.78 ± 0.78 ***

Values are presented as mean ± SEM; * *p* < 0.05; ** *p* < 0.01; *** *p* < 0.001 compared to cells with no treatment (control).

## Data Availability

Not applicable.

## Supplementary Materials

The following supporting information can be downloaded at: https://www.mdpi.com/article/10.3390/molecules27082500/s1, Figure S1: MS/MS spectra of fractions isolated from a *B. caapi* methanolic extract using semi-preparative C18 HPLC-DAD (230 nm). Specra were acquired by a QTOF spectrometer with ESI ionization QTOF: Quadrupole Time of Flight. ESI: Electrospray. A: F1 [M + H]^+^ *m*/*z* = 174.0923; B: F1 [M + H]^+^ *m*/*z*= 233,1294; C: F2 ([M + H]^+^ *m*/*z* = 353.1721); D: F3 ([M + H]^+^ *m*/*z* = 304.3012); F4 ([M + H]^+^ *m*/*z* = 188,1062; F5 ([M + H]^+^ *m*/*z* = 250.0799); Figure S2: Cytokine production by BV-2 cells after treatment for 2 h with (A) *Banisteriopsis caapi* extract, BC, and fractions (B) F1 and (C) F2 (1.4 to 182 µM). Mean ± SEM; * *p* < 0.05; ** *p* < 0.01 compared to cells with no treatment (control). *n* = 6 for control and *n* = 3 for treatments, except for F1 at 0.5 µg/mL and F2 at 32 µg/mL (*n* = 5).

## Sample Availability

Samples of the compounds are not available from the authors.
