# Supplementary material for: Components of Banisteriopsis caapi, a Plant Used in the Preparation of the Psychoactive Ayahuasca, Induce Anti-Inflammatory Effects in Microglial Cells
Source: Molecules. 2022 Apr 13;27(8):2500. doi: 10.3390/molecules27082500 (PMC9025580; doi:10.3390/molecules27082500)
Supplement: Supplementary file 1 [file molecules-27-02500-s001.zip › molecules-1646170-supplementary.pdf]

Components of *Banisteriopsis caapi*, a Plant Used in the Preparation of the Psychoactive Ayahuasca, Induce Anti-Inflammatory Effects in Microglial Cells

Beatriz Werneck Lopes Santos, Daniel Carneiro Moreira, Tatiana Karla dos Santos Borges, Eloisa Dutra Caldas*

University of Brasilia, Brasilia, DF, Brazil

*eloisa@unb.br

Supplementary materials

A: fraction F1

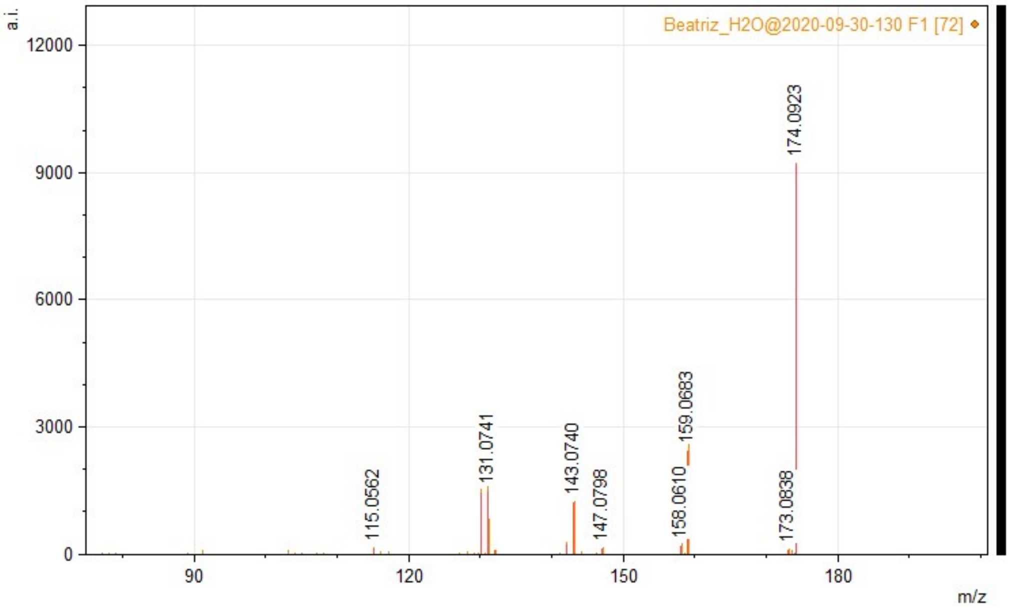

B: fraction F1

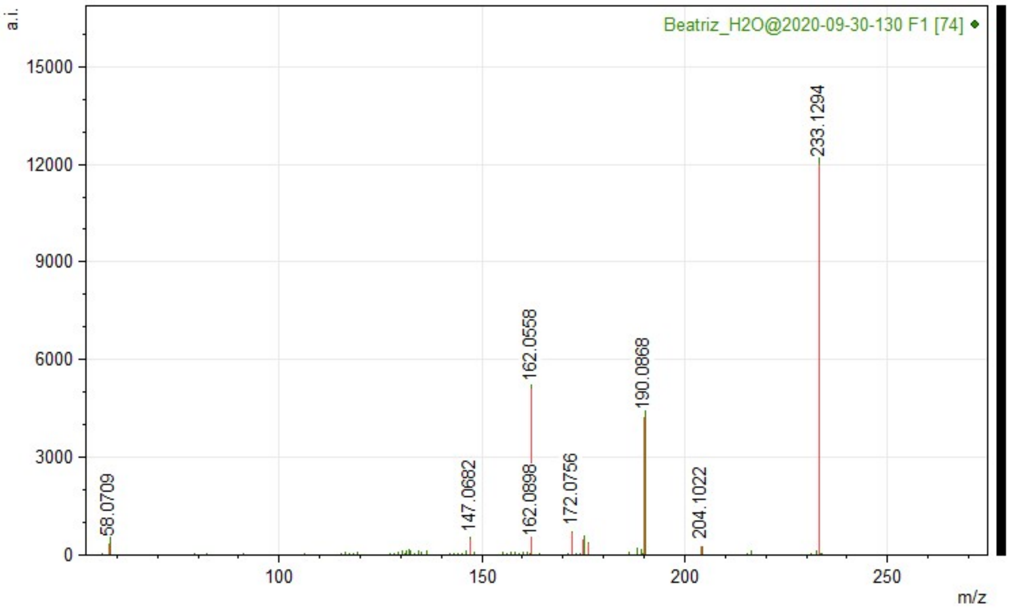

C: fraction F2

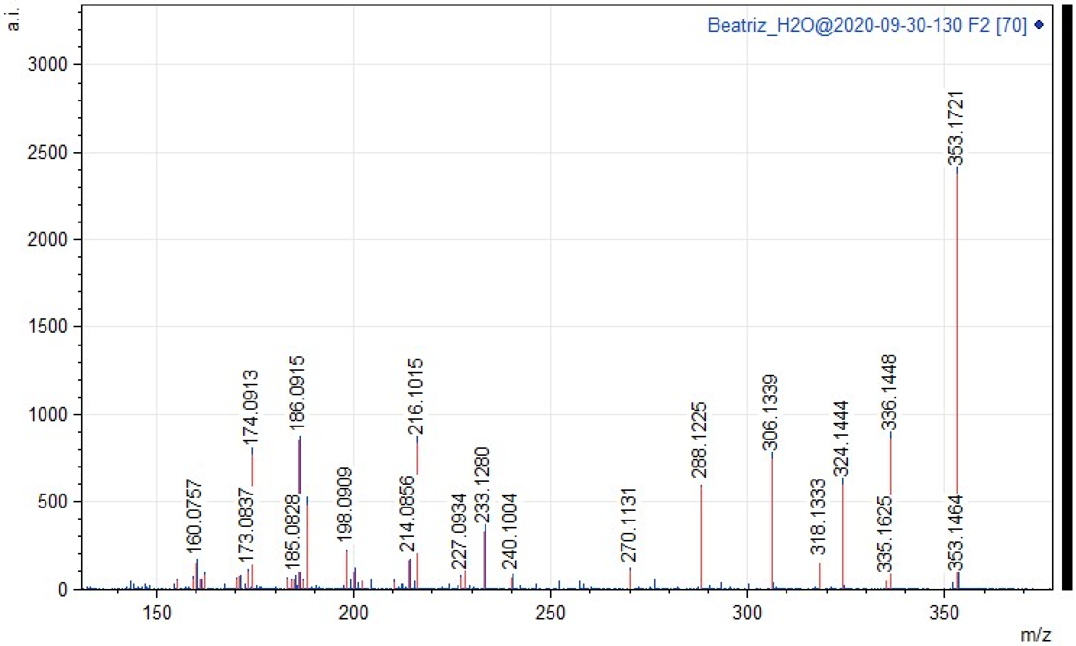

D: fraction F3

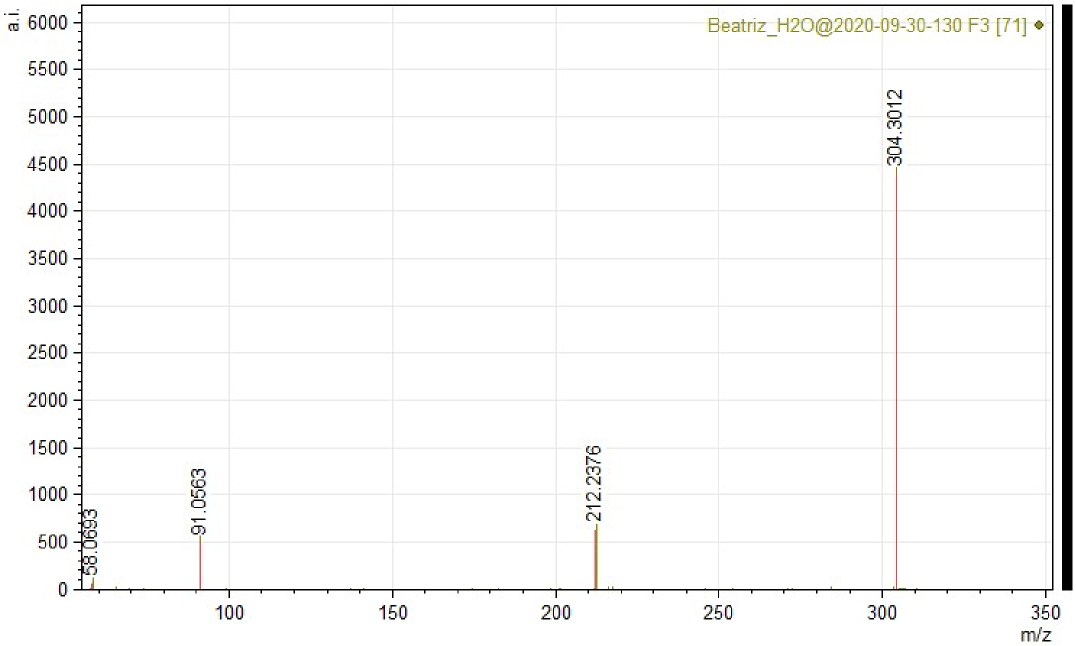

E: fraction F4

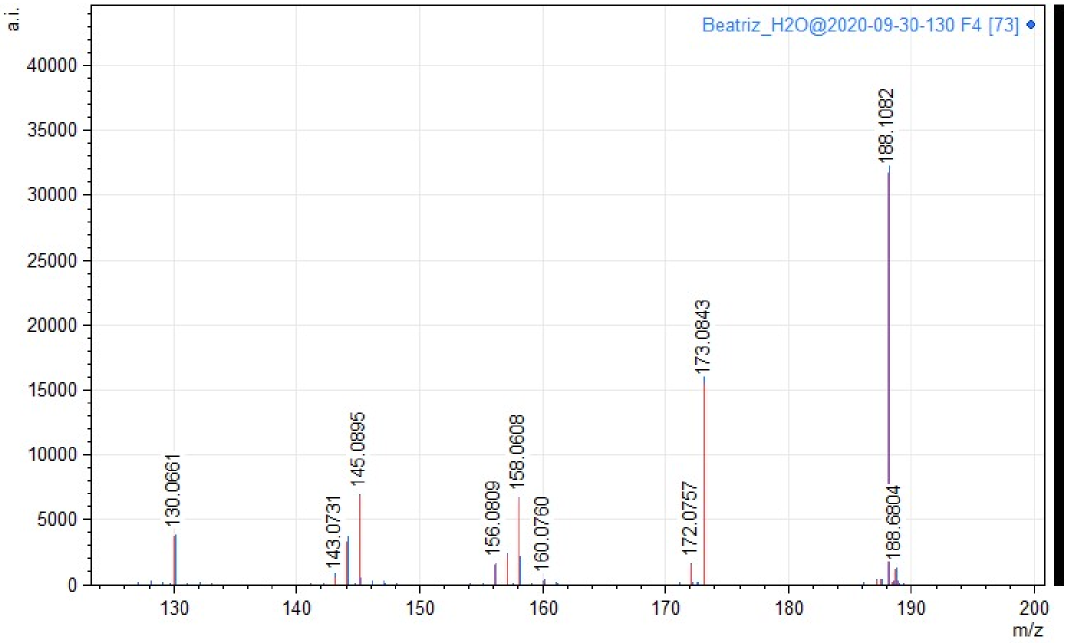

F: fraction F5

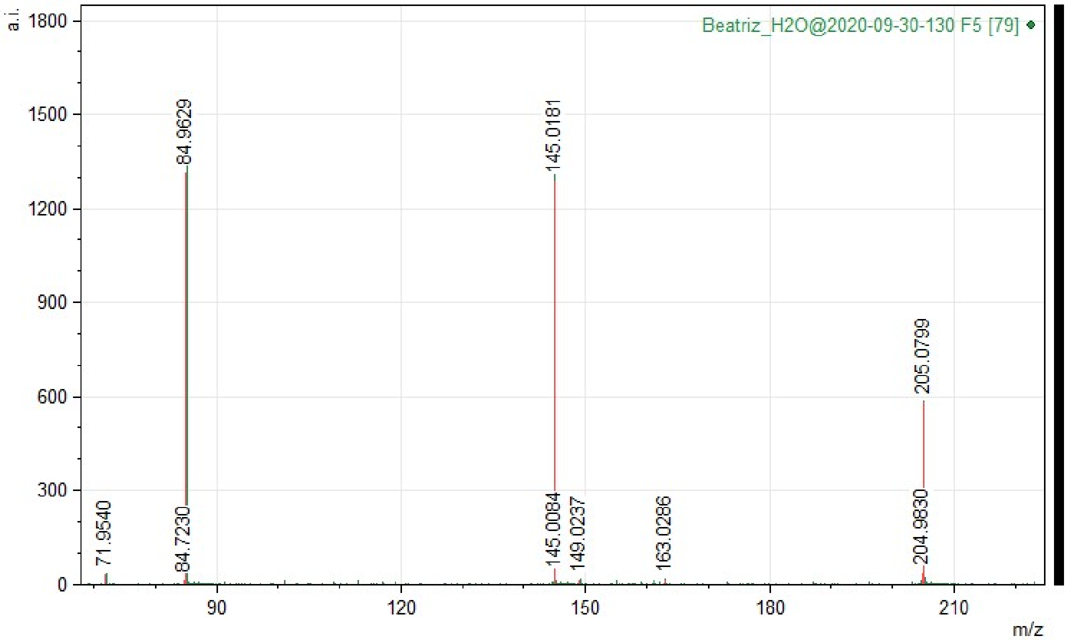

Figure S1: MS/MS spectra of fractions isolated from a *B. caapi* methanolic extract using semi-preparative C18 HPLC-DAD (230 nm). Specra were acquired by a QTOF spectrometer with ESI ionization QTOF: Quadrupole Time of Flight. ESI: Electrospray. A: F1 [M+H]+ m/z = 174.0923; B: F1 [M+H]+ m/z= 233,1294; C: F2 ([M+H]+ m/z = 353.1721); D: F3 ([M+H]+ m/z = 304.3012); F4 ([M+H]+ m/z = 188,1062; F5 ([M+H]+ m/z = 250.0799)

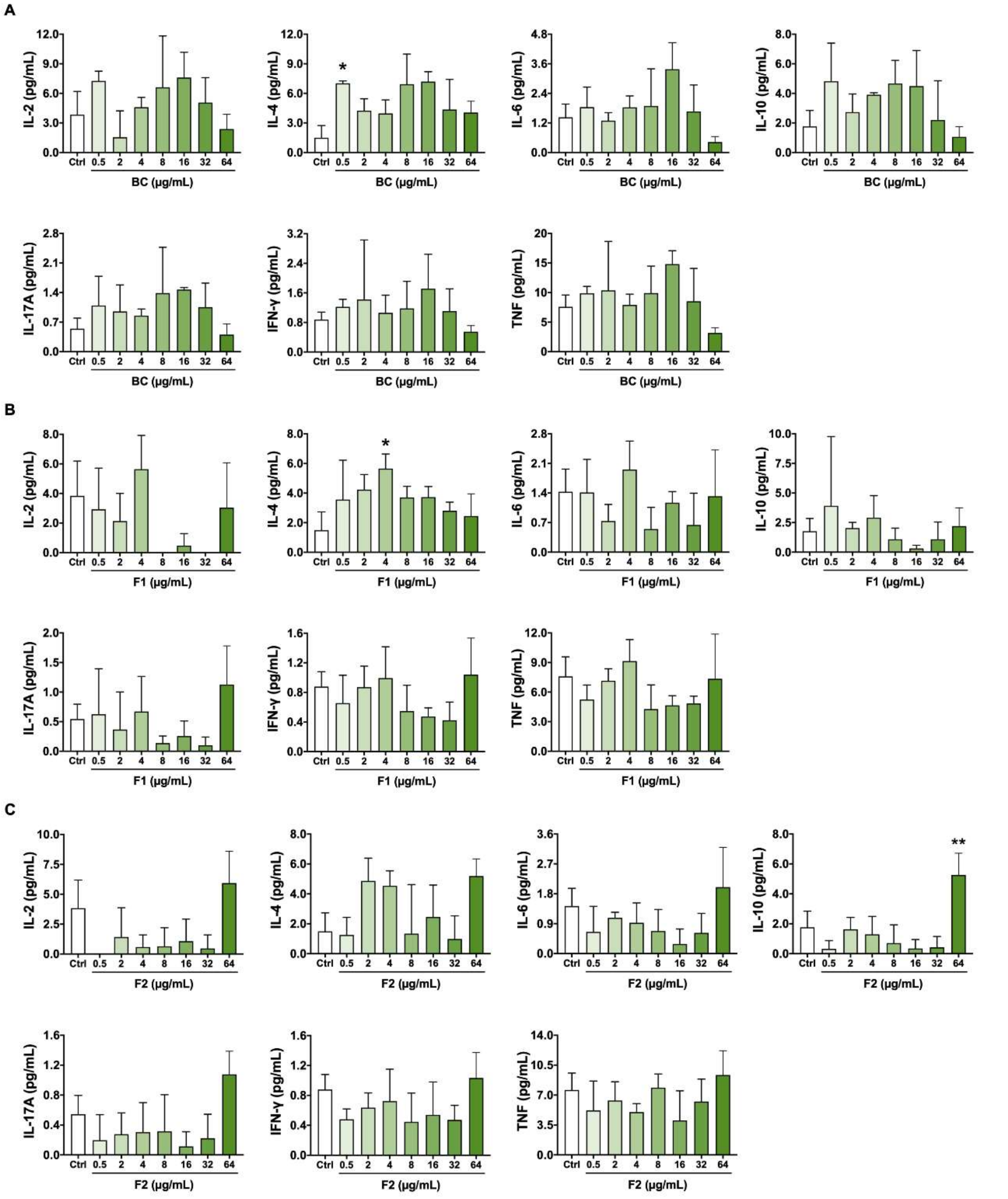

Figure S2. Cytokine production by BV-2 cells after treatment for 2 h with (A) *Banisteriopsis caapi* extract, BC, and fractions (B) F1 and (C) F2 (1.4 to 182 µM). Mean ± SEM; *$p<0.05$; **$p<0.01$ compared to cells with no treatment (control). n=6 for control and n=3 for treatments, except for F1 at 0.5 µg/mL and F2 at 32 µg/mL (n=5).
